# Capacitation-Induced Mitochondrial Activity Is Required for Sperm Fertilizing Ability in Mice by Modulating Hyperactivation

**DOI:** 10.3389/fcell.2021.767161

**Published:** 2021-10-26

**Authors:** María Milagros Giaccagli, Matías Daniel Gómez-Elías, Jael Dafne Herzfeld, Clara Isabel Marín-Briggiler, Patricia Sara Cuasnicú, Débora Juana Cohen, Vanina Gabriela Da Ros

**Affiliations:** ^1^Laboratorio de Mecanismos Moleculares de la Fertilización, Instituto de Biología y Medicina Experimental (IByME-CONICET), Buenos Aires, Argentina; ^2^Laboratorio de Biología Celular y Molecular de la Reproducción, Instituto de Biología y Medicina Experimental (IByME-CONICET), Buenos Aires, Argentina

**Keywords:** mitochondria, capacitation, hyperactivation, fertilization, sperm

## Abstract

To become fully competent to fertilize an egg, mammalian sperm undergo a series of functional changes within the female tract, known as capacitation, that require an adequate supply and management of energy. However, the contribution of each ATP generating pathway to sustain the capacitation-associated changes remains unclear. Based on this, we investigated the role of mitochondrial activity in the acquisition of sperm fertilizing ability during capacitation in mice. For this purpose, the dynamics of the mitochondrial membrane potential (MMP) was studied by flow cytometry with the probe tetramethylrhodamine ethyl ester (TMRE). We observed a time-dependent increase in MMP only in capacitated sperm as well as a specific staining with the probe in the flagellar region where mitochondria are confined. The MMP rise was prevented when sperm were exposed to the mitochondrial uncoupler carbonyl cyanide m-chlorophenyl hydrazine (CCCP) or the protein kinase A (PKA) inhibitor H89 during capacitation, indicating that MMP increase is dependent on capacitation and H89-sensitive events. Results showed that whereas nearly all motile sperm were TMRE positive, immotile cells were mostly TMRE negative, supporting an association between high MMP and sperm motility. Furthermore, CCCP treatment during capacitation did not affect PKA substrate and tyrosine phosphorylations but produced a decrease in hyperactivation measured by computer assisted sperm analysis (CASA), similar to that observed after H89 exposure. In addition, CCCP inhibited the *in vitro* sperm fertilizing ability without affecting cumulus penetration and gamete fusion, indicating that the hyperactivation supported by mitochondrial function is needed mainly for *zona pellucida* penetration. Finally, complementary *in vivo* fertilization experiments further demonstrated the fundamental role of mitochondrial activity for sperm function. Altogether, our results show the physiological relevance of mitochondrial functionality for sperm fertilization competence.

## Introduction

Mammalian fertilization is a complex process that involves different sequential interactions between the spermatozoon and the egg. As this interplay occurs in the oviduct, sperm must reach it from the semen deposit site in the vagina or uterus, depending on the species (Yanagimachi, 1994). During this transport, sperm experience a series of functional and structural modifications collectively known as capacitation (Chang, 1951; Austin, 1952). These changes are molecularly triggered by the entry of HCO_3_^–^ and Ca^2+^, which activate protein kinase A (PKA)-dependent signaling cascades leading to phosphorylation of proteins, increase in intracellular pH and hyperpolarization of the plasma membrane potential (reviewed in Puga Molina et al., 2018). Capacitation can be mimicked *in vitro* in a defined medium containing HCO_3_^–^, Ca^2+^, a cholesterol acceptor (commonly serum albumin) and energy sources (Yanagimachi, 1994). The two functional consequences of this process are the ability to undergo acrosome reaction, essential for sperm to penetrate and fuse with the egg (Yanagimachi, 1994), and the development of hyperactivation, critical for sperm to swim through the oviductal fluid, detach from the isthmus reservoir and penetrate the envelopes that surround the egg (Demott and Suarez, 1992; Suarez and Dai, 1992; Yanagimachi, 1994; Stauss et al., 1995; Brukman et al., 2016). Taken together, the acquisition of the capacitation status is mandatory for the cells to become fertilization competent.

Sperm motility disorders are one of most relevant causes of male infertility (Nowicka-bauer and Nixon, 2020; Shahrokhi et al., 2020; Tu et al., 2020). Understanding the cellular and molecular mechanisms involved in flagellar movement is required to improve diagnosis and treatment of the associated pathologies. Although the etiology of these disorders is known only in few cases, it could be associated with structural or functional sperm defects, such as dysregulation of specific signaling pathways or energy production (Nowicka-bauer and Nixon, 2020; Shahrokhi et al., 2020; Tu et al., 2020). In view of this, research has been carried out to elucidate the individual contribution of each energy metabolic pathways, glycolysis and oxidative phosphorylation (OXPHOS), occurring in the sperm tail (Fawcett, 1975; Bunch et al., 1998; Eddy, 2006; Krisfalusi et al., 2006; Balbach et al., 2020), to sustain motility. However, the results obtained so far in different species are controversial. In humans, whereas several studies revealed that glucose, the main glycolytic substrate, plays a key role in supplying ATP for motility, others showed the importance of OXPHOS for motility and sperm function (reviewed in Ruiz-Pesini et al., 2007; Boguenet et al., 2021). This controversy might be partially attributed to differences in the experimental conditions used in each case. In addition, it can also be due to an often disregard for the facts that glycolysis both requires ATP to start the process, contrary to OXPHOS, and it is usually a prerequisite for OXPHOS (Ramalho-Santos et al., 2009; Barbagallo et al., 2020). In this sense, a functional association between these two pathways has been recently described in sperm (Tourmente et al., 2015; Balbach et al., 2020). Therefore, besides not reaching a consensus, there is no direct evidence showing an association between these energy metabolic pathways and sperm fertilizing ability in humans due to ethical limitations, reinforcing research using animal models.

In mice, knockout studies showed that glycolysis (Miki et al., 2004; Odet et al., 2008; Danshina et al., 2010), rather than OXPHOS (Narisawa et al., 2002; Nayernia et al., 2002), is essential for sustaining sperm motility and male fertility. In addition, whereas several glycolytic (i.e., glucose, fructose, and mannose) and non-glycolytic (i.e., lactate and pyruvate) substrates maintained sperm motility (Mukai and Okuno, 2004; Goodson et al., 2012), only glucose and mannose were able to support hyperactivation (Goodson et al., 2012). Despite this, using an extracellular flux analyzer, it has recently been shown that mouse sperm enhance both glycolysis and OXPHOS to sustain the energy demand increase during capacitation (Balbach et al., 2020). However, in that case, capacitation was induced through a pharmacological stimulation of PKA, opening the possibility that these energy providing pathways could be differently regulated under physiological conditions. Therefore, fertilization assays to evaluate whether the provenance of ATP is relevant for acquisition of motility and fertilization competence remain necessary.

Considering the above findings and that mitochondrial metabolism is superior to glycolysis in terms of ATP production, the aim of this study was to determine the role of mitochondrial activity in the acquisition of sperm fertilizing ability during capacitation in mice. Here, we show the dynamics of the mitochondrial membrane potential (MMP; also referred in the literature as ΔΨm) during capacitation, which reflects the cellular capacity to produce ATP by OXPHOS and, therefore, it is used as an indicator of mitochondrial activity (Nicholls and Ward, 2000). Our study was performed with the cationic lipophilic dye tetramethylrhodamine, ethyl ester (TMRE) that had not been widely used for evaluation of sperm quality (Marchetti et al., 2004; Losano et al., 2017), despite several of its attractive characteristics, such as low mitochondrial toxicity, its single-channel fluorescence is simple to analyze and it can be combined with other probes for multiparametric staining (Nicholls and Ward, 2000; Marchetti et al., 2004). In addition, we analyzed the relevance of mitochondrial function not only for hyperactivation but also for *in vitro* and *in vivo* sperm fertilizing ability.

## Materials and Methods

### Animals

Hybrid (C57BL/6xBALB/c) F1 male (age: 3–6 months) and female (age: 45 days–4 months) mice were housed in the animal facility at IBYME-CONICET (Buenos Aires, Argentina) and maintained with food and water *ad libitum* in a temperature-controlled room (21–23°C) with light:dark (12:12 h, lights on: 7:00 AM) cycle. Approval for the study protocol was obtained from the Institutional Animal Care and Use Committee of Instituto de Biología y Medicina Experimental (N° 08/2021). Experiments involving animals were performed in accordance with the Guide for Care and Use of Laboratory Animals published by the National Institutes of Health.

### Reagents

Reagents and chemicals were purchased from Sigma-Aldrich (St Louis, MO), unless otherwise indicated.

### Sperm Capacitation

Mouse sperm were recovered by incising the cauda epididymis in 300 μl of capacitation medium containing 99.3 mM NaCl, 2.7 mM KCl, 1.8 mM CaCl_2_.2H_2_O, 0.3 mM Na_2_H_2_PO_4_.2H_2_0, 0.5 mM MgCl_2_.2H_2_O, 25 mM NaHCO_3_, 5.6 mM glucose, 24.4 mM lactate and 0.5 mM pyruvate, and supplemented with 0.3% (w/v) bovine serum albumin (BSA), pH: 7.3–7.5 (“swim-out”) (Da Ros et al., 2008). Aliquots of the suspension were added to 300 μl of capacitation medium containing either carbonyl cyanide 3-chlorophenylhydrazone (CCCP), H89 (Cayman Chemical, Ann Arbor, MI) or dimethyl sulphoxide (DMSO; Baker, Phillipsburg, NJ), as vehicle (< 1% v/v), to give a final concentration of 5–10 × 10^6^ cells/ml. Sperm suspensions were then incubated for 90 min at 37°C in an atmosphere with 5% (v/v) CO_2_ in air.

### Mitochondrial Membrane Potential Determination

For MMP analysis by flow cytometry, the “swim out” procedure was carried out in a BSA-free medium. As this medium does not support mouse sperm capacitation (Visconti et al., 1995, 1999), it is considered to be non-capacitating. Aliquots of the sperm suspension were added to 200 μl of BSA-free medium or of capacitation medium containing CCCP (concentration range: 5–80 μM), H89 (20 μM) or DMSO. After different time periods of incubation (0, 40, 70 min) sperm were loaded with 0.1 μM TMRE (Invitrogen Carlsbad, CA) and incubated for 20 additional minutes. Samples were washed to remove the excess of probe by centrifugation at 725 ×*g* for 3 min, resuspended in the BSA-free medium, and exposed without permeabilization to 0.02 μg/ml 4′,6-diamidine-2′-phenylindole dihydrochloride (DAPI; Invitrogen) just before measurement to assess cell viability (see experiment annotation example in Lee et al., 2008). Fluorescence was detected using a BD FACSCantoTM II analyzer (BD Biosciences, East Rutherford, NJ) following the manufacturer’s indications. DAPI and TMRE fluorescence was collected using the Pacific Blue (450/50) and PE (585/42) filters, respectively. One technical replicate (20000 measured events) was performed for each treatment in each independent experiment. After acquisition, fluorescence compensation and data analysis were performed by FlowJo 10 software (FlowJo LLC, Ashland, OR). The overall gating strategy used is shown in the corresponding Figure and its legend. Results are presented as mean fluorescence intensity (MFI) for TMRE and percentage of cells showing high TMRE signal.

For localization studies, sperm treated with CCCP (20 μM) or DMSO were loaded with 0.1 μM TMRE and 15 μg/ml Hoechst 33342 (Invitrogen), and incubated to complete the 90 min period. Micrographs were obtained from living sperm samples seeded in polylysine (0.1 mg/ml) coverslips and observed under an Olympus IX83 Spinning Disk microscope (Olympus Corp., Tokyo, Japan) (× 600).

### Protein Phosphorylation Assessment

After capacitation in the presence of CCCP (concentration range: 5–60 μM) or DMSO, protein phosphorylation was assessed as previously reported (Da Ros et al., 2008; Weigel Muñoz et al., 2018). Sperm suspensions were washed with PBS, resuspended in Laemmli sample buffer (Laemmli, 1970), then boiled for 5 min and centrifuged at 2.000 ×*g*. The supernatants were boiled again in the presence of 70 mM 2-β-mercaptoethanol, and solubilized proteins (corresponding to 5 × 10^6^ sperm/lane) were separated by SDS-PAGE (7.5% polyacrylamide) and transferred onto nitrocellulose. After blocking with 2% skim milk in PBS-Tween, the membranes were probed with either anti-phospho-PKA substrate (1:1000; clone 9624, Cell Signaling Technology, Danvers MA) or anti-phosphotyrosine antibody (1:1000; clone 4G10; Merck MilliPore, Burlington, MA). Next, the membranes were incubated with the corresponding peroxidase-conjugated secondary antibody (1:4000; Vector Laboratories, Burlingame, CA). The immunoreactive proteins were detected by ECL Western blotting kit (Thermo Fisher, Waltham, MA) and images captured with G:BOX GENI (Syngene, Synoptics Ltd, Cambridge, England) according to the manufacturer’s instructions. For quantification, the pixels of each lane in the images were calculated using the ImageJ software.^1^ Each value was relativized to the one of the phospho-hexokinase (116 kDa) band of the same lane, as this protein is constitutively Tyr-phosphorylated (Kalab et al., 1994; Visconti et al., 1996), and then normalized to the control lane (CAP, see figure legend) of each blot. For this purpose, the phospho-PKA substrate blots were stripped and further probed with the anti-phosphotyrosine antibody to detect the phospho-hexokinase band in the same samples.

### Simultaneous Evaluation of Motility and Mitochondrial Membrane Potential

Twenty min before the end of capacitation, sperm were loaded with 0.1 μM TMRE and 15 μg/ml Hoechst 33342 (Invitrogen), and incubated to complete the 90 min period. Samples were then washed, resuspended in fresh medium, mounted in pre-warmed slides and observed under a Nikon Optiphot microscope (Nikon, Tokyo, Japan) equipped with epifluorescence optics (× 500). Sperm were scored motile or immotile and as TMRE positive or negative depending on the presence of a bright red staining in the midpiece of the flagellum.

### Motility Assessment by Computer Assisted Sperm Analysis

After 90 min-capacitation in the presence of CCCP (concentration range: 10–60 μM), H89 (20 μM) or DMSO, sperm aliquots (15 μl) were placed between pre-warmed slides and cover slips (22 × 22 mm) to create a chamber with 30 μm depth, and were examined at 37°C using Sperm Class Analyzer^®^ system (SCA v.6.2.0.1., Microptic SL, Barcelona, Spain). Drifting was set in 25 μm/s. At least 200 sperm distributed in a minimum of 10 different microscope fields were evaluated (30 frames acquired at 60 Hz for each measurement). The following parameters were assessed: curvilinear velocity (VCL, μm/s), straight line velocity (VSL, μm/s), average path velocity (VAP, μm/s), linearity (LIN,%), straightness (STR,%), wobble (WOB,%), amplitude of lateral head displacement (ALH, μm) and beat cross frequency (BCF, Hz). Sperm were considered hyperactivated when presenting VCL ≥ 271 μm/s, LIN < 50% and ALH ≥ 3.5 μm. These custom cutoffs were selected based on our experience (Brukman et al., 2016) and previously reported recommendations (Bray et al., 2005).

### *In vitro* Fertilization Assays

Gamete interaction assays were carried out as previously reported (Da Ros et al., 2008). Briefly, female mice were superovulated by an injection of eCG (5 UI, Syntex, Buenos Aires, Argentina), followed by hCG (5 UI, Syntex) 48 h later. Cumulus-oocyte complexes (COCs) were collected from the oviducts 13–14 h after hCG administration and pooled. When needed, cumulus cells were removed by incubating the COCs in 0.3 mg/ml hyaluronidase (type IV) for 3–5 min. In some cases, the *zona pellucida* (ZP) was dissolved by treating the eggs with acid Tyrode solution (pH 2.5) for 10–20 s (Nicolson et al., 1975). Sperm were incubated for 90 min in the capacitation medium with different concentrations of CCCP (concentration range: 20–60 μM) or DMSO. After that, sperm were washed, and resuspended in a fresh medium for insemination.

COCs and ZP-intact eggs were inseminated with a final concentration of 1–5 × 10^5^ cells/ml and gametes co-incubated for 3 h at 37°C in an atmosphere of 5% (v/v) CO_2_ in air. Eggs were then fixed with 2% (w/v) paraformaldehyde in PBS, washed, stained with 10 μg/ml Hoechst 33342, mounted on slides and finally analyzed under the epifluorescence microscope (× 250). For fusion assays, ZP-free eggs were inseminated with a final concentration of 1–5 × 10^4^ cells/ml and gametes co-incubated for 1 h under the same incubation conditions as stated above. Eggs were then fixed with 2.5% glutaraldehyde (Baker), stained with 1% aceto-carmine solution and observed under the microscope (× 400). In all cases, eggs were considered fertilized when at least one decondensing sperm nucleus or two pronuclei were observed in the egg cytoplasm.

### Cumulus Penetration Assay

After sperm incubation in the presence of CCCP (concentration range: 5–60 μM), H89 (20 μM) or DMSO, cumulus penetration assays were performed as previously described (Ernesto et al., 2015). Briefly, sperm were stained with 5 μg/ml Hoechst 33342, and used to inseminate the COCs (final concentration: 1–2.5 × 10^4^ sperm/ml). Gametes were co-incubated for 15 min at 37°C in an atmosphere of 5% (v/v) CO_2_ in air. COCs were then washed, fixed with 2% (w/v) paraformaldehyde and the number of sperm within the cumulus was determined under the epifluorescence microscope (× 250).

### Intrauterine Insemination

Intrauterine insemination assays were performed as previously described (Curci et al., 2021). Briefly, female mice were superovulated by an injection of eCG, followed by hCG 46 h later. Nine h later, females were anesthetized with ketamine (100 mg/kg, Holliday- Scott SA, Buenos Aires, Argentina)—xilacine (10 mg/kg, Richmond Vet Farma SA, Buenos Aires, Argentina), and both uterine horns were surgically exposed. Then, sperm suspensions (1–10 × 10^7^ sperm/ml) preincubated with different concentrations of CCCP (range: 20–60 μM) or DMSO for 20 min were injected into the uterine horns using one for CCCP-treated sperm and the contralateral for control sperm. After surgery, females were placed on a warm pad until complete recovery. Fifteen h later, COCs were collected from the *ampulla*, and incubated in KSOM medium (Erbach et al., 1994), scoring the percentage of 2-cell embryos 24 h later. Embryos were then transferred to a fresh KSOM medium drop to evaluate the development to the blastocyst stage on day 4 after insemination.

### Statistical Analysis

Calculations were performed using the Prism 80.0 software (GraphPad Software, La Jolla, CA). Data was analyzed by one- or two-way analysis of the variance (ANOVA) after checking data normality (Shapiro-Wilk test) and homoscedasticity (Spearman’s test for two-way ANOVA or Brown-Forsythe test for one-way ANOVA). Transformations were performed when assumptions were violated. One-way ANOVA followed by Fisher’s LSD post-test was used for determining the effect of CCCP on MMP (except % of sperm TMRE+), kinematic parameters (except linearity index), hyperactivation, and *in vitro* and *in vivo* sperm fertilizing ability. Two-way ANOVA followed by Fisher’s LSD post-test was used for determining the MMP dynamics during capacitation and the simultaneous evaluation of MMP and motility. Data represents the mean ± SEM of independent experiments. In cases where the assumptions remained unfulfilled, the non-parametric Kruskal-Wallis test followed by Dunn’s post-test was used for determining the effect of CCCP on MMP (% of sperm TMRE+), sperm cumulus penetration ability and linearity index. Data represents the median with interquartile range. In all cases, differences were considered significant at a level of *p* < 0.05.

## Results

### Assessment of Mitochondrial Membrane Potential Dynamics During Sperm Capacitation

**FIGURE 1:**
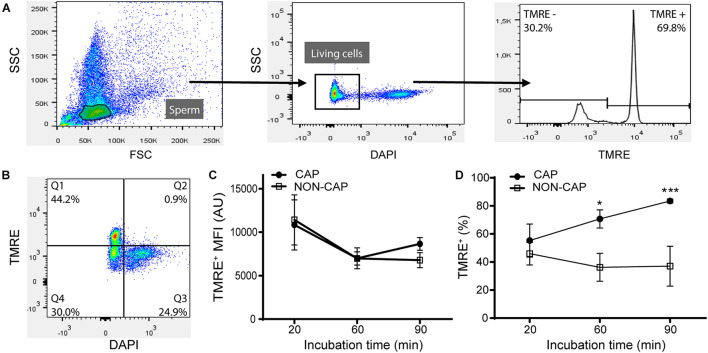
Determination of mitochondrial membrane potential by flow cytometry during mouse sperm capacitation. Epididymal mouse sperm were incubated under non-capacitating or capacitating conditions, loaded with TMRE, and fluorescence was measured by flow cytometry. **(A)** Gating strategy used in the analysis: sperm were selected on a forward (FSC) and side scatter (SSC) plot (left panel) and, then, DAPI negative (living) cells (center panel) were further gated to determine the percentage of TMRE+ and TMRE− cells (right panel). Representative data of capacitated sperm is shown. **(B)** A representative image of a TMRE and DAPI scatter plot showing that only living cells present TMRE staining (Q1). **(C)** Mean fluorescence intensity (MFI) from capacitated (CAP) and non-capacitated (NON-CAP) TMRE + sperm at different incubation times. **(D)** Percentage of CAP and NON-CAP TMRE + sperm at different incubation times. Results are expressed as mean ± SEM. *n* = 5. *p < 0.05, ***p < 0.001.

To study the role of mitochondrial activity in the acquisition of sperm fertilizing ability in mice, we first evaluated its dynamics during capacitation. For these experiments, we measured MMP in sperm using the probe TMRE that emits high fluorescence in living cells when it is sequestered by active mitochondria with high MMP (Nicholls and Ward, 2000). As this dye had not been previously used in mouse sperm, initial experiments were carried out to set up the proper concentration and incubation time for MMP determination (data not shown). After this, epididymal sperm were incubated under capacitating or non-capacitating (BSA-free) conditions for different periods of time, then loaded with TMRE, and finally analyzed for fluorescence intensity by flow cytometry (Figure 1A). Results showed that, as expected, only living cells presented TMRE staining (DAPI negative cells) (Figure 1B). In addition, the MFI of the TMRE positive population remained similar under both incubation conditions and constant over time (Figure 1C). On the other hand, the percentage of TMRE positive cells gradually increased during capacitation, tripling the value of the non-capacitated ones at the end of the incubation (3.2 ± 0.7 times, *n* = 5) (Figure 1D). The fact that at time = 20 min no differences in MMP values were observed between incubations with or without BSA, and that the time-dependent increase in MMP dynamics along capacitation was observed even in the presence of BSA (Figure 1D), argues against the possibility that the difference in TMRE between non-capacitating and capacitating conditions is only caused by a different dye solubility or loading due to the presence of BSA. Altogether, these data support that during capacitation, cells undergo mitochondrial activation.

**FIGURE 2:**
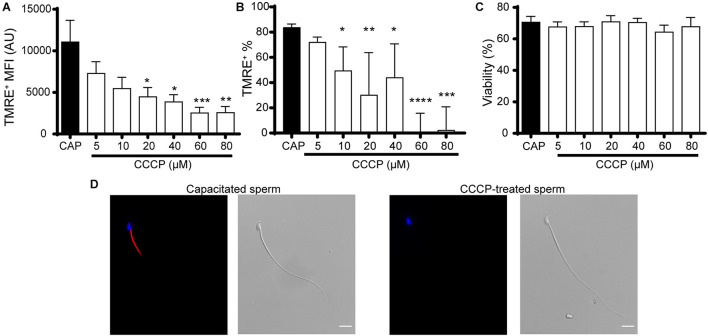
Validation of TMRE as a probe for the analysis of mitochondrial membrane potential in mouse sperm. Epididymal sperm were incubated in the capacitation medium containing CCCP (5–80 μM) or DMSO (capacitated sperm, CAP), loaded with TMRE, and at 90 min of total incubation fluorescence was measured by flow cytometry. The following parameters were scored: **(A)** Mean fluorescence intensity (MFI). **(B)** Percentage of TMRE+ sperm. **(C)** Percentage of sperm viability. Results are expressed as mean ± SEM in **(A,C)** and median with interquartile range in **(B)**. In all cases, at least 3 independent experiments were performed. *p < 0.05, **p < 0.01, ***p < 0.001, ****p < 0.0001. **(D)** Representative fluorescence and phase-contrast microscopy images showing TMRE (red) and Hoechst 33342 (blue) staining patterns of capacitated (left) and CCCP-treated (right) sperm. *n* = 3. Bar = 10 μm.

To further validate the use of TMRE to measure MMP in mouse sperm, flow cytometry experiments were repeated on sperm incubated during capacitation with different concentrations of the mitochondrial OXPHOS uncoupler, CCCP. Under these conditions, a dose-dependent decrease in both the MFI of the TMRE positive population (Figure 2A) and the percentage of TMRE positive cells (Figure 2B) was observed, which was significant from 10 μM CCCP. Viability controls using DAPI revealed that CCCP did not affect the percentage of living cells at any of the tested concentrations (Figure 2C). Subsequent fluorescence microscopy studies in capacitated cells showed TMRE signal only in the midpiece of the flagellum (Figure 2D left panels), consistent with the localization of mitochondria (Fawcett, 1975; Eddy, 2006; Gervasi et al., 2018). Accordingly, in CCCP-treated samples, sperm without TMRE staining were observed (Figure 2D right panels).

**FIGURE 3:**
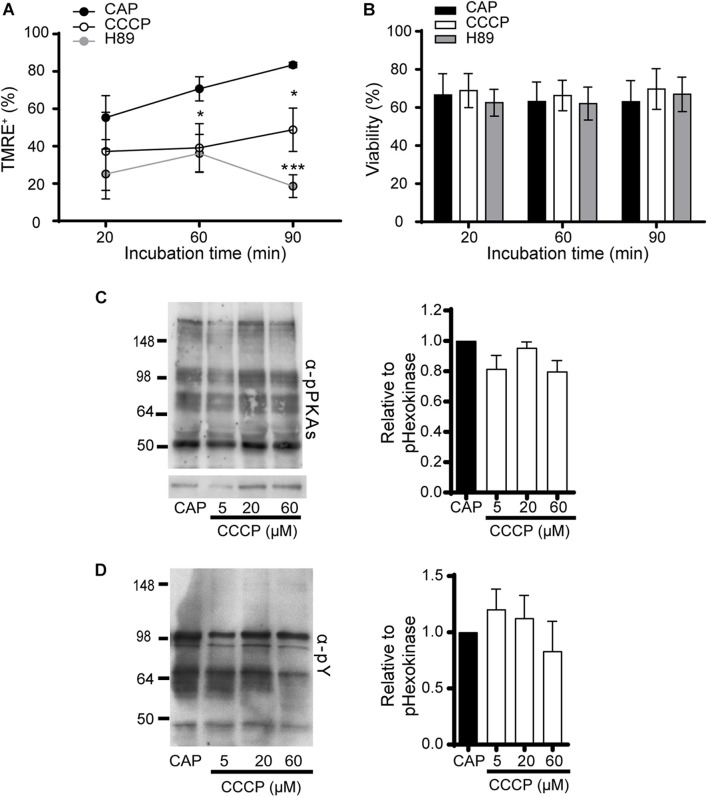
Association between the increase in mitochondrial activity and capacitation in mouse sperm. **(A,B)** Epididymal sperm were incubated in the capacitation medium containing CCCP (20 μM), H89 (20 μM) or DMSO (capacitated sperm, CAP), loaded with TMRE, and fluorescence was measured by flow cytometry. The following parameters were scored: **(A)** Percentage of sperm exhibiting TMRE + signal. **(B)** Percentage of viability from experiments shown in **(A)**. Results are expressed as mean ± SEM. *n* = 5. *p < 0.05, ***p < 0.001. **(C,D)** Epididymal sperm were incubated in the capacitation medium containing CCCP (5–60 μM) or DMSO (capacitated sperm, CAP), and analyzed by Western blotting for phosphorylation **(C)** in PKA substrates (α-pPKAs) and **(D)** in tyrosine residues (α-pY). Representative blots are shown on the left. Right panels correspond to quantification graphs of each phosphorylation normalized to CAP. At least 3 independent experiments were performed.

As the above results supporting the use of TMRE to assess sperm MMP dynamics, revealed an increase in this parameter during capacitation, two different strategies were undertaken to further analyze the association between mitochondrial activity and capacitation. In the first case, we evaluated whether the increase in MMP depends on capacitation-associated signaling pathways. For this purpose, sperm were incubated in the capacitation medium in the presence of H89, which blocks the capacitation-induced PKA signaling cascade, and MMP was determined by flow cytometry. Sperm incubated under capacitating conditions in the presence of CCCP or vehicle (capacitated sperm) were used as controls. Contrary to capacitated sperm, cells exposed to H89 or CCCP showed no increase in the percentage of cells exhibiting TMRE staining at the end of incubation (Figure 3A), with similar percentages of sperm viability among groups and time periods (Figure 3B). As a second approach, we investigated whether the increase in MMP was required for the occurrence of capacitation-associated signaling pathways leading to protein phosphorylation. To this end, sperm were incubated under capacitating conditions with different concentrations of CCCP, and the phosphorylation of proteins normally observed during capacitation (Visconti et al., 1995; Krapf et al., 2010) was assessed by Western blotting. Results showed no statistically significant differences in the capacitation-associated increase in either PKA substrates or tyrosine phosphorylations at any of the conditions tested (Figures 3C,D). Altogether, these results show that the increase in MMP is dependent on capacitation and H89-sensitive events, and that the PKA signaling cascade is not affected by mitochondrial disruption.

### Relevance of Capacitation-Induced Mitochondrial Activity for Motility

**FIGURE 4:**
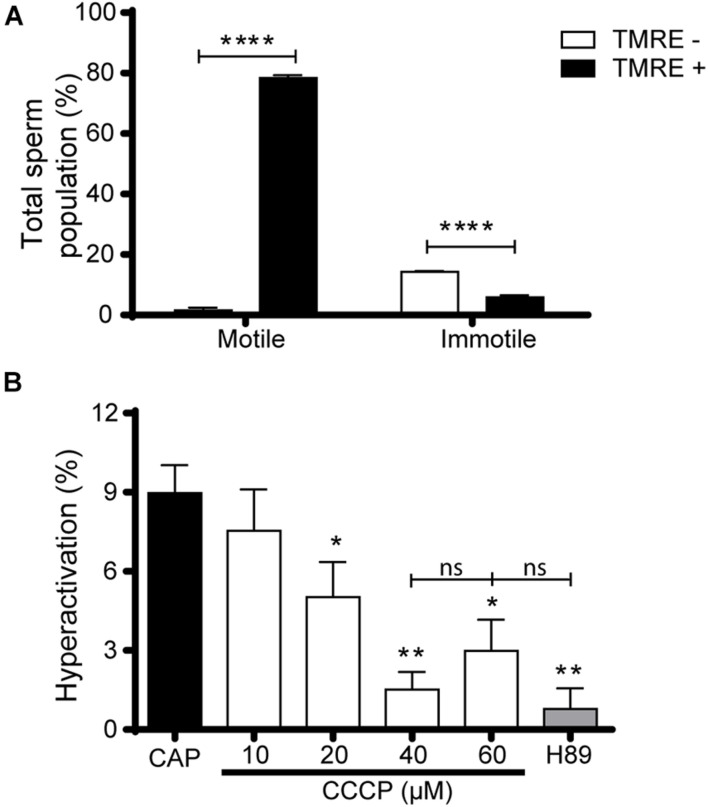
Association between the increase in mitochondrial activity and motility in mouse sperm. **(A)** Epididymal sperm were incubated under capacitating conditions, loaded with TMRE, and motility as well as TMRE staining were simultaneously analyzed by microscopy to calculate the percentage of motile and immotile sperm with or without TMRE signal (TMRE+ and TMRE-, respectively). *n* = 3 **(B)** Epididymal sperm were incubated in the capacitation medium containing CCCP (10–60 μM), or H89 (20 μM) or DMSO (capacitated sperm, CAP), and the percentage of hyperactivation in motile sperm determined by CASA. At least 4 independent experiments were performed. In all cases, results are expressed as mean ± SEM; *p < 0.05, **p < 0.01, ****p < 0.0001.

In order to unveil an association between mitochondrial activity and motility in capacitated sperm, we simultaneously evaluated both variables in the same cell by microscopy. Whereas motility was subjectively recorded as motile or immotile in a bright field, high MMP was evaluated with TMRE by fluorescent staining. Results revealed that nearly all cells within the motile capacitated sperm population (98.1 ± 1.1%, *n* = 3) exhibited TMRE labeling (Figure 4A). In addition, immotile cells were mostly TMRE negative (71.1 ± 2.8%, *n* = 3) (Figure 4A). These observations support a strong association between high MMP and motility in capacitated sperm. Furthermore, objective analysis of motility by Computer assisted sperm analysis (CASA) showed that sperm treated with CCCP during capacitation exhibited a significant decrease in several of the kinematic parameters (Supplementary Table 1) as well as in the percentage of hyperactivation from 20 μM CCCP (Figure 4B) compared to control capacitated cells. Of note, there was no statistically significant difference between the effect produced by CCCP at 40 or 60 μM and H89 on hyperactivation (Figure 4B). Altogether, these results argue in favor of a role of mitochondrial activity in the development of hyperactivation during capacitation.

### Relevance of Capacitation-Induced Mitochondrial Activity for *in vitro* Sperm Fertilizing Ability

**FIGURE 5:**
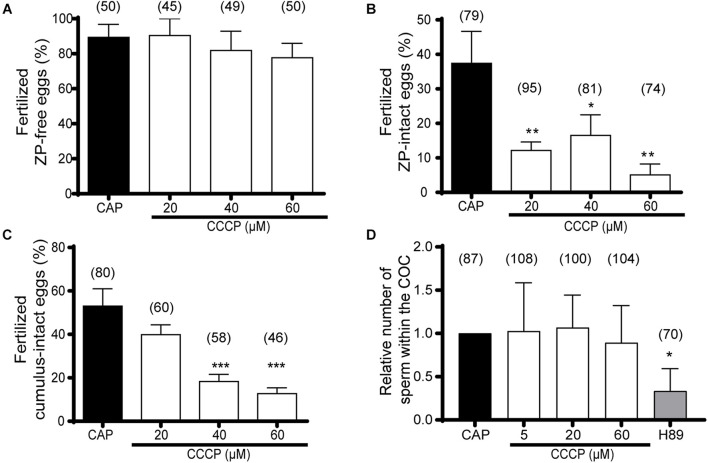
Relevance of mitochondrial activity on mouse sperm *in vitro* fertilizing ability. **(A–C)** Epididymal sperm were incubated in the capacitation medium containing CCCP (20–60 μM) or DMSO (capacitated sperm, CAP), and used to inseminate **(A)** ZP-free eggs (*n* = 4), **(B)** ZP-intact eggs (*n* = 5) or **(C)** cumulus-intact eggs (*n* = 4). The percentage of fertilized eggs was determined in all cases. **(D)** Epididymal sperm were incubated in the capacitation medium containing CCCP (5–60 μM), H89 (20 μM), or DMSO (capacitated sperm, CAP), loaded with Hoechst 33342, and used to inseminate cumulus-intact eggs. After 15 min, the number of bright sperm heads within the cumulus matrix was determined. Results representing the relative numbers to the capacitated group are shown (*n* = 6). In brackets is indicated the total number of analyzed eggs per treatment. Results are expressed as mean ± SEM for **(A–C)** and median with interquartile range for **(D)**. *p < 0.05, **p < 0.01, ***p < 0.001.

To fully understand whether the rise in mitochondrial activity during capacitation is necessary for sperm to become fertilization competent, *in vitro* fertilization studies were carried out. For these experiments, sperm incubated under capacitating conditions with different concentrations of CCCP were washed, resuspended in fresh medium, and used to inseminate either ZP-free eggs, ZP-intact eggs or eggs surrounded by both the cumulus and the ZP (COCs). Results obtained using ZP-free eggs to evaluate gamete fusion showed similar fertilization rates among all treatments (Figure 5A). In contrast, in both approaches using eggs with ZP (with or without cumulus cells), a significant decrease in fertilization rates was observed for CCCP-treated sperm compared to controls without CCCP (Figures 5B,C). Interestingly, whereas for ZP-intact assays 20 μM CCCP was enough to produce a significantly negative effect, in cumulus-intact assays 40 μM was needed, supporting the already proposed beneficial effect of cumulus cells for capacitation and/or fertilization (Yanagimachi, 1994; Da Ros et al., 2008). Altogether, these results indicate that mitochondrial function is required for the acquisition of sperm fertilizing ability in mice in a step previous to gamete fusion. Considering our observations showing that mitochondrial activity is necessary for hyperactivation and that this type of motility is required for penetration of the egg envelopes (Suarez and Dai, 1992; Yanagimachi, 1994; Stauss et al., 1995; Brukman et al., 2016), we next investigated whether the fertilization impairments observed were due to a failure in egg coat penetration. For this purpose, we performed cumulus penetration assays where CCCP-exposed sperm and controls (capacitated sperm and H89-treated sperm) were stained with Hoechst 33342 and used to inseminate COCs, recording the number of fluorescent sperm heads inside the *cumulus oophorus* 15 min later. Of note, the CCCP and H89 concentrations used were those that had produced an inhibitory effect on hyperactivation (see Figure 4B). As shown in Figure 5D, whereas few sperm were capable of penetrating the cumulus mass when incubated in the presence of H89, higher and similar numbers were observed for those incubated with CCCP as well as for capacitated control cells. These results do not support a role of mitochondria in the ability of sperm to penetrate the *cumulus oophorus*, indicating that hyperactivation induced by mitochondrial activity is mainly needed for ZP penetration.

### Relevance of Capacitation-Induced Mitochondrial Activity for *in vivo* Sperm Fertilizing Ability

**FIGURE 6:**
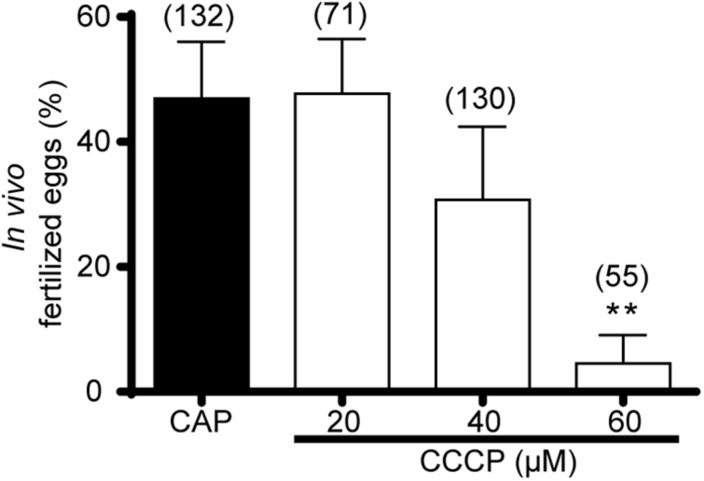
Relevance of mitochondrial activity on mouse sperm *in vivo* fertilizing ability. Epididymal sperm in a non-capacitating concentration were incubated in the capacitation medium containing CCCP (20–60 μM) or DMSO (capacitated sperm, CAP), and used to inseminate superovulated females. After 15 h, COCs were collected from the *ampulla*, and incubated in KSOM medium, scoring the percentage of 2-cell embryos 24 h later. Results are expressed as mean ± SEM. At least 4 independent experiments were performed in which one or more females per treatment were included. In brackets is indicated the total number of analyzed eggs per treatment. **p < 0.01.

Based on the above *in vitro* observations, we next explored the relevance of mitochondrial function *in vivo*. As a proof of concept of this hypothesis, intrauterine inseminations in superovulated females were performed with sperm pre-treated with different concentrations of CCCP, and the fertilization rates were then analyzed. Results revealed a significant progressive decrease in the *in vivo* percentage of fertilized eggs as CCCP concentration increased, with a significant effect at 60 μM (Figure 6). For the few fertilized eggs obtained from CCCP-treated sperm, embryonic development was allowed to continue *in vitro*, observing normal blastocysts (data not shown). Altogether, these results show the key role of mitochondria function for not only the *in vitro* but also *in vivo* fertilizing ability of mouse sperm.

## Discussion

In the current study, mouse sperm mitochondrial activity was studied in depth in order to evaluate its dynamics during capacitation and its role for the acquisition of sperm fertilizing ability. Our principal contribution relies on the fact that mouse sperm capacitation is accompanied by a rise in mitochondrial activity which is required for hyperactivation and penetration of the egg envelope, likely the ZP rather than the *cumulus oophorus*. Complementary *in vivo* fertilization experiments further demonstrated the relevance of mitochondrial activity for sperm function, emphasizing the physiological importance of mitochondrial functionality for sperm fertilization competence.

Early studies of mitochondrial activity in mouse sperm were focused on O_2_ consumption reporting a constant or a declined rate during capacitation (Boell, 1985; Fraser and Lane, 1987). On the other hand, recent works have shown an increase during capacitation in both OXPHOS, determined by an extracellular flux analyzer (Balbach et al., 2020), and MMP, analyzed by flow cytometry using JC-1 (Yang et al., 2020), a probe widely used despite its complexities and false results (Perry et al., 2011). However, none of these techniques allows the simultaneous determination of mitochondrial activity and viability in a single cell. In this sense, our study provides several advantages in the approach designed to overcome this limitation. First, we measured MMP by using TMRE, a probe that has never been used before in mouse sperm despite enabling multiparametric staining (Nicholls and Ward, 2000; Marchetti et al., 2004). This characteristic of the dye led us to assess MMP and viability in single sperm by flow cytometry. Second, our approach allowed us to perform these measurements in the presence of HCO_3_^–^, the physiological activator of capacitation. This is particularly relevant when considering recent reports showing an increase in glycolysis and OXPHOS during mouse sperm capacitation induced by pharmacological PKA activators (Balbach et al., 2020), although a lower glucose consumption was then observed under those conditions (Hidalgo et al., 2020). Therefore, our study is the first to evaluate MMP dynamics in single living sperm undergoing capacitation in physiological conditions.

Our results revealed a gradual increase in the number of sperm cells with high MMP during capacitation, in contraposition to the constant number observed when cells were incubated under non-capacitating conditions. The statistical difference between both conditions was observed around 1 h, suggesting that the rise in mitochondrial function in mouse sperm might be related to a mid or late event of the capacitation process. Interestingly, the lack of BSA in the presence of HCO_3_^–^ in the capacitation medium (BSA-free or non-capacitating medium) precluded that increase, possibly due to the described role of this protein in the activation of HCO_3_^–^/PKA signaling pathway (Osheroff et al., 1999; Visconti et al., 1999). This effect is different to that observed under the same incubation conditions when glucose consumption was the endpoint measurement (Hidalgo et al., 2020), revealing different regulatory mechanisms for each energy metabolic pathway, and thus the importance of directly measuring MMP in mouse sperm. The fact that the TMRE fluorescence intensity of the positive cells did not change with the incubation conditions suggests that mitochondrial activation during capacitation is an “all-or-nothing” process within each sperm.

Specificity of the TMRE staining was further confirmed by the restricted localization of its fluorescence to the midpiece of the flagellum, the region in which mitochondria are confined in sperm (Fawcett, 1975; Eddy, 2006; Gervasi et al., 2018). Finally, the observation that the addition of CCCP to the capacitation medium abrogated the rise in MMP, both in flow cytometry and microscopy studies, validated the use of TMRE for MMP evaluation in mouse sperm. Of note, the collapse of mitochondrial activity induced by CCCP did not compromise sperm viability, at least during the time period analyzed, although mitochondrial activity disturbance is often associated with apoptosis in other cell types (Boguenet et al., 2021). Altogether, our observations support a role of mitochondrial function along the capacitation process. Therefore, besides the increase in intracellular pH and hyperpolarization of the plasma membrane potential, among others (reviewed in Puga Molina et al., 2018), the MMP rise represents another hallmark of capacitation that could be used as a new biomarker of this process in mouse sperm. Moreover, the successful setup of the TMRE measurement will undoubtedly be useful to further understand sperm physiology by analyzing MMP and other capacitation-induced parameters (i.e., pH increase), simultaneously, in single-cell approaches.

Our experiments aimed to investigate the crosstalk between energy metabolic and signaling pathways during capacitation showed that the observed rise in mitochondrial functionality required at least the activation of the PKA signaling pathway. These results are consistent with the mentioned report showing an increase in mitochondrial activity after the downstream stimulation of HCO_3_^–^-induced sAC (Balbach et al., 2020), jointly supporting an involvement of the HCO_3_^–^/sAC/PKA pathway in mitochondrial activity during mouse sperm capacitation. The other approach used to study this crosstalk showed that there is no significant effect of CCCP on PKA substrate phosphorylation, an expected result considering that this phosphorylation is an early event of capacitation (Krapf et al., 2010; Battistone et al., 2013) whereas the increase in mitochondrial activity was observed around 1 h of capacitation. In addition, similar results were obtained for tyrosine phosphorylation, in line with previous reports (Travis et al., 2001; Goodson et al., 2012; Balbach et al., 2020), indicating that mitochondrial function may not be essential to sustain phosphorylation of sperm proteins. Therefore, this led us to conclude that other energy metabolic pathways should be sufficient to support these phosphorylations.

Considering the conflict around the relevance of mitochondrial activity for mouse sperm motility (Mukai and Okuno, 2004; Goodson et al., 2012; Takei et al., 2014), we assessed simultaneously, in the same cell, if the increase in MMP was linked to the motility status of the capacitated cells. Our findings showing that motile sperm exclusively exhibited TMRE staining whereas the immotile cells were predominantly TMRE negatives, support a strong association between mitochondrial function and motility in mouse capacitated sperm. The very small number of immotile sperm displaying TMRE signal might be attributed to residual fluorescence of previously motile sperm. To our knowledge, this is the first study that simultaneously monitors both the occurrence of an energy metabolic pathway and a functional sperm parameter in a single cell subjected to capacitation. Of note, although previous studies showed no association between mitochondrial function and sperm motility (Mukai and Okuno, 2004; Takei et al., 2014), no references were made to whether sperm were incubated or not under capacitating conditions, particularly about the presence of BSA in the medium, which seems to be required for mitochondrial activity according to our results. Moreover, when we objectively analyzed motility after capacitation by CASA, several kinematic parameters as well as the percentage of hyperactivated sperm decreased in the groups exposed to CCCP in comparison to the capacitated control, supporting a role for mitochondrial function in the acquisition and/or maintenance of hyperactivation. Our data showing that sperm mitochondrial disruption results in normal protein phosphorylation with a reduced hyperactivation, reveals that this type of motility implies more than the activation of the phosphorylation pathway, including molecular mechanisms that depend on mitochondrial activity (Ramalho-Santos et al., 2009). Their precise contribution to hyperactivation requires further investigation. Interestingly, Goodson et al. (2012) showed that the addition of glucose or mannose, contrary to pyruvate and lactate, in the capacitation medium supported hyperactivation, suggesting a role for glycolysis over mitochondrial metabolism in this type of motility. A possible explanation to merge their and our results might be that hyperactivation depends on both pathways as suggested by the facts that: (1) hyperactivation is sustained in the presence of glucose (Goodson et al., 2012), condition in which both glycolysis and mitochondria are active, considering the recent reported link between both metabolic pathways (Tourmente et al., 2015; Balbach et al., 2020), and (2) hyperactivation is diminished when only one of these pathways is active (Goodson et al., 2012 and present results), i.e., in the presence of pyruvate only mitochondria are functional, and in the presence of CCCP only glycolysis is active.

Having observed a role of mitochondrial functionality in capacitation, we then evaluated whether this was relevant for the acquisition of sperm fertilizing ability. *In vitro* gamete fusion assays revealed that mitochondrial activity during capacitation was not required for sperm to fuse with the egg, reinforcing previous observations precluding the requirement of hyperactivation for this step of gamete interaction (Yanagimachi, 1994; Ren et al., 2001; Xie et al., 2006). On the other hand, *in vitro* fertilization studies demonstrated the need of mitochondrial function during capacitation for sperm to penetrate the egg coats. In line with our results, Balbach et al. (2020) have recently reported higher fertilization rates of cumulus-intact eggs when sperm were capacitated in the presence of both glucose and pyruvate than the mere presence of only one of them, suggesting that both glycolysis and mitochondrial metabolism are contributing, possibly to a different extent, to the development of not only hyperactivation but also the sperm fertilizing ability. In this regard, and considering that hyperactivation is required for egg envelope penetration (Suarez and Dai, 1992; Yanagimachi, 1994; Stauss et al., 1995; Brukman et al., 2016), our results support the idea that the low fertilization rates of sperm exposed to CCCP were linked to the observed defects in hyperactivation. The fact that CCCP-treated sperm were able to penetrate the *cumulus oophorus* supports a role of mitochondrial activity-induced hyperactivation mainly in the ZP penetration step. Interestingly, H89-exposed sperm, exhibiting similar hyperactivation defects than CCCP-treated sperm, produced lower cumulus penetration rates. Therefore, the flagellar movement assigned in both cases as hyperactivation by CASA was functionally different, suggesting that physiologically relevant motility still cannot be measured by current methods and, therefore, the development of alternative overcoming approaches for its determination, such as 3D high-resolution flagellar tracking (Nandagiri et al., 2021), might be needed. Alternatively, the requirement of hyperactivation, as determined by CASA, for sperm penetration of the cumulus matrix might need some revision (Suarez and Dai, 1992; Brukman et al., 2016).

Taking into account that the *in vitro* capacitation conditions may differ from those encountered by sperm in their transit through the female reproductive tract, in particular considering that the availability of nutrients and its concentrations *in vivo* are poorly defined, performing *in vivo* studies was critical to determine the physiological relevance of our *in vitro* findings. Our results from intrauterine insemination experiments revealed the need of mitochondrial function for sperm to fertilize the egg *in vivo* as well as the availability of oxidizable substrates in the female reproductive tract. Although the use of CCCP in these experiments is regarded as a proof of concept to study mitochondrial function under physiological conditions, this type of *in vivo* approach is unique in terms of the potentiality of discovering similarities and disparities between *in vitro* and *in vivo* capacitation. In this sense, different CCCP concentrations between both conditions were needed to obtain a significant effect in the fertilizing ability of sperm. Besides this, we could not assess whether the *in vivo* effect was attributable to the one observed *in vitro* in ZP penetration or, additionally, to another hyperactivation-dependent event such as swimming through the oviductal fluid (Demott and Suarez, 1992; Suarez and Dai, 1992; Yanagimachi, 1994; Stauss et al., 1995; Brukman et al., 2016) due to the lack of appropriate tools to study sperm migration. In line with this, an association between mitochondrial dysfunctionality and male infertility as a result of a diminished sperm motility has been reported for several knockout models (VPS13A, Tppp2, Gykl1, and Gk2) (Chen et al., 2017; Nagata et al., 2018; Zhu et al., 2019).

It has been postulated that whereas glycolysis is used for activities requiring quick and local increases of ATP, OXPHOS is a more efficient source of ATP over time (Zecchin et al., 2015). Therefore, considering the high demand on ATP sperm have in their long journey through the female reproductive tract to accomplish fertilization in the *ampulla*, it is tempting to speculate that *in vivo* sperm may utilize both metabolic pathways in response to the different extracellular energy substrates to produce ATP. In view of this, more innovative strategies are still needed to be developed in order to fully understand how sperm metabolism could shift *in vivo* between glycolysis and OXPHOS.

In humans, MMP has been postulated as a predictive marker of sperm fertilization ability in both natural conception and *in vitro* fertilization (reviewed in Ramalho-Santos et al., 2009; Boguenet et al., 2021). However, the precise role of mitochondrial activity in human sperm is hampered primarily by ethical issues concerning research with human eggs. In the present study, we explored this in the mouse model which, in spite of presenting some differences in the molecular mechanisms underlying capacitation compared to human (Puga Molina et al., 2018; Boguenet et al., 2021), is the best approach that can be used to study gamete interaction *in vitro* and *in vivo*. In view of this, our findings demonstrating that *in vivo* sperm fertilizing ability is dependent on mitochondrial activity could help to understand sperm physiology and might serve as the basis for future studies focusing on the mitochondria as a target for contraception development and/or for diagnosis and treatment of fertility disorders.

## Data Availability Statement

The raw data supporting the conclusions of this article will be made available by the authors, without undue reservation.

## Ethics Statement

The animal study was reviewed and approved by Institutional Animal Care and Use Committee of Instituto de Biología y Medicina Experimental.

## Author Contributions

MG, MG-E, and JH performed all experiments. CM-B collaborated with CASA evaluation. MG, DC, and VD designed the experiments, analyzed the results, and wrote the manuscript. PC contributed intellectual content in the experimental design and the discussion of results. All authors read, corrected, and approved the final manuscript.

## Conflict of Interest

The authors declare that the research was conducted in the absence of any commercial or financial relationships that could be construed as a potential conflict of interest.

## Publisher’s Note

All claims expressed in this article are solely those of the authors and do not necessarily represent those of their affiliated organizations, or those of the publisher, the editors and the reviewers. Any product that may be evaluated in this article, or claim that may be made by its manufacturer, is not guaranteed or endorsed by the publisher.

## Footnotes

1
http://imagej.nih.gov/ij

## Abbreviations

BSA: bovine serum albumin
CASA: computer-assisted sperm analysis
CCCP: carbonyl cyanide 3-chlorophenylhydrazone
COCs: cumulus–oocyte complexes
DAPI: 4′,6-diamidine-2′-phenylindole dihydrochloride
DMSO: dimethyl sulfoxide
MFI: mean fluorescence intensity
MMP: mitochondrial membrane potential
OXPHOS: oxidative phosphorylation
PKA: protein kinase A
TMRE: tetramethylrhodamine, ethyl ester
ZP: *zona pellucida*.
