# Supplementary material for: Capacitation-Induced Mitochondrial Activity Is Required for Sperm Fertilizing Ability in Mice by Modulating Hyperactivation
Source: Front Cell Dev Biol. 2021 Oct 26;9:767161. doi: 10.3389/fcell.2021.767161 (PMC8576324; doi:10.3389/fcell.2021.767161)
Supplement: Supplementary file 1 [file Table_1.docx]

**Supplementary Material**

**Supplementary Table 1. Effect of mitochondrial activity disruption on motility assessed by CASA.**

| **Kinematic parameters** | **CAP** | **CCCP (µM)** | | | | **H89** |
| --- | --- | --- | --- | --- | --- | --- |
|  |  | **10** | **20** | **40** | **60** |  |
| **VCL (μm/s)** | 168.4 ± 5.6 | 165.5 ± 12.3 | 145.1 ± 12 | 124.6 ± 7.9 (**) | 133.7 ± 7 (*) | 102.1 ± 18.7 (***) |
| **VSL (μm/s)** | 42.4 ± 1.7 | 42.7 ± 2.7 | 33.2 ± 4.1 | 27.9 ± 3.6 (*) | 32.0 ± 2.4 (*) | 24.2 ± 8 (**) |
| **VAP (μm/s)** | 105.9 ± 3.5 | 106.1 ± 9.5 | 90.4 ± 8 | 81.9 ± 6.7 (*) | 88.5 ± 3.7 (*) | 62.3 ± 15.3 (***) |
| **LIN (%)** | 25.0 (2.2) | 26.1 (4) | 23.4 (10) | 20.5 (7.1) | 24.3 (7) | 22.5 (23.4) |
| **STR (%)** | 39.0 ± 1 | 39.3 ± 1.4 | 35.6 ± 2.2 | 32.6 ± 2.5 | 34.9 ± 1.8 | 35.9 ± 3.9 |
| **WOB (%)** | 61.8 ± 1.2 | 62.1 ± 1.9 | 60.3 ± 2.6 | 64.0 ± 1.6 | 65.4 ± 1.5 | 52.7 ± 11.5 |
| **ALH (μm)** | 3.5 ± 0.1 | 3.4 ± 0.2 | 3.0 ± 0.2 | 2.6 ± 0.1 (**) | 2.7 ± 0.1 (**) | 2.2 ± 0.4 (***) |
| **BCF (Hz)** | 10.5 ± 0.2 | 10.3 ± 0.2 | 9.8 ± 0.7 | 9.9 ± 0.6 | 10.8 ± 0.3 | 7.9 ± 1.7 |

Epididymal sperm were incubated in the capacitation medium containing CCCP (10-60 μM), H89 (20 μM) or DMSO (capacitated sperm, CAP), and kinematic parameters were measured by computer-assisted sperm analysis (CASA). VCL, curvilinear velocity; VSL, straight line velocity; VAP, average path velocity; LIN, linearity; STR, straightness; ALH, amplitude of lateral head; BCF, beat frequency. In all cases, at least 4 independent experiments were performed. Results are expressed as mean ± SEM, except for LIN expressed as median (interquartile range); **p*<0.05, ***p*<0.01, ****p*<0.001.
